# Artificial intelligence enabled histological scoring in ulcerative colitis: Are we ready yet?

**DOI:** 10.1002/ueg2.12579

**Published:** 2024-05-18

**Authors:** Marietta Iacucci, Yasuharu Maeda, Subrata Ghosh

**Affiliations:** ^1^ APC Microbiome Ireland College of Medicine and Health University College of Cork Cork Ireland

**Keywords:** diagnosis, disease clearance, endoscopy, histological healing, IBD, indices, inflammatory bowel disease, mucosal, remission, target

Ulcerative colitis (UC) disease activity assessment poses challenges to clinicians and pathologists alike due to its complex nature and the subjective interpretation of histological findings. Existing histological indices rely heavily on human subjective judgment, which, while invaluable, introduces variability and potential bias in disease assessment.^1^, ^2^ Histological remission is emphasized as a “deeper” healing and disease clearance than endoscopic remission, reducing the risks of future relapse, colectomy, and colorectal cancer progression in clinical practice. Histological remission has also been adopted as an endpoint for clinical trials, often combined with endoscopic endpoint. A manual central reading has been introduced to overcome the limitations of inter‐pathologist variabilities in participating in international trials. However, it is expensive and time‐consuming while still not perfect regarding observer variability. Integrating artificial intelligence (AI) and digital pathology in this realm offers a promising solution by providing a more automated objective and reproducible evaluation of histological disease activity and healing.^3^

In the Journal's current issue, Peyrin‐Biroulet et al.^4^ present their innovative AI‐enabled scoring system based on the Nancy histological index (NHI). To address the challenge of the NHI being composed of several pathological findings, the authors integrated four AI neural networks to establish an algorithm that predicts a five‐level NHI of 0–4. The AI tool's performance was determined to correlate highly with pathologists' assessments, potentially promising its utilization in daily practice and, eventually, trials. Such application could reduce the necessity for extensive training and resource allocation, eradicate the subjectivity inherent in pathologists' evaluations and facilitate the assessment of disease severity for informed treatment decisions and prediction of outcomes.

AI‐enabled histological scoring systems employ machine learning algorithms trained on vast datasets of annotated digitally scanned images to recognize patterns, anomalies, and the extent of inflammation characteristic of UC. These systems can analyze histological slides with precision and consistency unattainable by human evaluators. The benefits are objective, accurate disease assessment and quantification of disease severity,^5^ enhancing predictive power of disease progression^6^ and response to therapy,^7^ and ultimately, a more personalized approach to patient management^8^ ‐ fulfilling “The mantra of the right therapy for the right patient.”

However, several issues need to be addressed before integrating AI‐enabled scoring based on digital pathology into clinical practice and clinical trials. Firstly, developing robust and reliable AI models requires extensive datasets that accurately represent the demographic and geographic diversity of the population affected by UC and require precise annotation protocols to minimize bias. It is essential to minimize prevailing variation and ascertain international standardization of digital hematoxylin and Eosin (H&E) histology specimens and rigorously report audit metrics. Recently, a novel active learning‐based digital pathology protocol for precise and objective annotation has been developed. It offers a novel approach for clinical trials and practices to alleviate the burden and reduce annotation bias, thereby improving histological assessment in UC.^9^ Additionally, standardization in interpreting AI diagnostic outputs by humans is imperative. The transparency of these systems in their assessment processes is critical, as AI's “black box” nature may engender skepticism amongst clinicians and patients. Ensuring the explainability of AI decisions is paramount in cultivating trust among healthcare professionals and patients and is a prerequisite for regulatory approval. Nonetheless, implementing such technology necessitates a multidisciplinary approach involving engineers, informaticians, clinicians, and pathologists integral to annotating and interpreting histological data.

Unfortunately, colonoscopy AI, which has already been clinically introduced, has been reported to only sometimes improve endoscopists' diagnostic accuracy as expected.^10^, ^11^ Training healthcare professionals to work alongside AI and interpret its findings is essential for successfully adopting these systems.

In conclusion, AI‐enabled scoring systems for measuring histological disease activity in UC represent a significant step forward in managing this complex disease. However, realizing this potential will require overcoming substantial hurdles, including data diversity, system transparency, and interdisciplinary collaboration. As we move forward, it is crucial to address these challenges head‐on, ensuring that AI is a valuable tool in the fight against UC rather than a complete replacement for the nuanced judgment of experienced medical professionals. This article, reported by Peyrin‐Biroulet et al.,^4^ is undoubtedly a pathway for humans and AI to create partnerships to fight against UC.

## CONFLICT OF INTEREST STATEMENT

None of the authors have a conflict of interest related to this manuscript.

## Data Availability

Data sharing not applicable to this article as no datasets were generated or analyzed during the current study.

## References

[1] Nardone OM , Iacucci M , Villanacci V , Peyrin‐Biroulet L , Ghosh S , Danese S , et al. Real‐world use of endoscopic and histological indices in ulcerative colitis: results of a global survey. United European Gastroenterol J. 2023;11(6):514–519. 10.1002/ueg2.12423 PMC10337739 37323091

[2] Feakins R , Borralho Nunes P , Driessen A , Gordon IO , Zidar N , Baldin P , et al. Definitions of histological abnormalities in inflammatory bowel disease: an ECCO position paper. J Crohns Colitis. 2024;18(2):175–191. 10.1093/ecco-jcc/jjad142 37607017 PMC10896637

[3] Gui X , Bazarova A , Del Amor R , Vieth M , de Hertogh G , Villanacci V , et al. PICaSSO Histologic Remission Index (PHRI) in ulcerative colitis: developing a novel simplified histological score for monitoring mucosal healing and predicting clinical outcomes and its applicability in an artificial intelligence system. Gut. 2022;71(5):889–898. 10.1136/gutjnl-2021-326376 35173041 PMC8995819

[4] Peyrin‐Biroulet L , Adsul S , Stancati A , Dehmeshki J , Kubassova O . An artificial intelligence–driven scoring system to measure histological disease activity in ulcerative colitis. United European Gastroenterol J. 2024. 10.1002/ueg2.12562 PMC11485311 38590110

[5] Ohara J , Nemoto T , Maeda Y , Ogata N , Kudo SE , Yamochi T . Deep learning‐based automated quantification of goblet cell mucus using histological images as a predictor of clinical relapse of ulcerative colitis with endoscopic remission. J Gastroenterol. 2022;57(12):962–970. 10.1007/s00535-022-01924-1 36184701

[6] Iacucci M , Parigi TL , Del Amor R , Meseguer P , Mandelli G , Bozzola A , et al. Artificial intelligence enabled histological prediction of remission or activity and clinical outcomes in ulcerative colitis. Gastroenterology. 2023;164(7):1180–1188.e2. 10.1053/j.gastro.2023.02.031 36871598

[7] Liu X , Prasath S , Siddiqui I , Walters TD , Denson LA , Consortium P , et al. Machine learning‐based prediction of pediatric ulcerative colitis treatment response using diagnostic histopathology. Gastroenterology. 2024;166(5):921–924.e4. 10.1053/j.gastro.2024.01.033 38309631 PMC11836768

[8] Iacucci M , Maeda Y , Ghosh S . A baby step or a real giant stride: histomic enabled by artificial intelligence to predict treatment response in pediatric patients with ulcerative colitis. Gastroenterology. 2024;166(5):730–732. 10.1053/j.gastro.2024.03.004 38460608

[9] Santacroce G , Meseguer P , Zammarchi I , Del Amor R , Hayes B , Crotty R , et al. P406 a novel active learning‐based digital pathology protocol annotation for histologic assessment in Ulcerative Colitis using PICaSSO Histologic Remission Index (PHRI). J Crohn's Colitis. 2024;18(Suppl_1):i843–i844. 10.1093/ecco-jcc/jjad212.0536

[10] Barua I , Wieszczy P , Kudo SE , Misawa M , Holme Ø , Gulati S , et al. Real‐time artificial intelligence–based optical diagnosis of neoplastic polyps during colonoscopy. NEJM Evid. 2022;1(6). 10.1056/evidoa2200003 38319238

[11] Djinbachian R , Haumesser C , Taghiakbari M , Pohl H , Barkun A , Sidani S , et al. Autonomous artificial intelligence vs artificial intelligence‐assisted human optical diagnosis of colorectal polyps: a randomized controlled trial. Gastroenterology. 2024. 10.1053/j.gastro.2024.01.044 38331204

